# The association between diabetes coexisting with low levels of high-density lipoprotein cholesterol and peritoneal dialysis-related peritonitis

**DOI:** 10.1186/s13098-022-00832-x

**Published:** 2022-04-29

**Authors:** Rui Zhang, Xing Zhang, Xingming Tang, Liwen Tang, Sijia Shang, Xiaoyang Wang, Yueqiang Wen, Xiaoran Feng, Qian Zhou, Ning Su, Yajuan Huang

**Affiliations:** 1 Department of Nephrology, The Sixth Affiliated Hospital of Sun Yat-Sen University, Guangzhou, 510655 China; 2 Department of Nephrology, The Affiliated Tung Wah Hospital of Sun Yat-Sen University, Dongguan, China; 3 Department of Nephrology, The First Affiliated Hospital of Zhengzhou University, Zhengzhou Univeristy, ZhengZhou, China; 4 Department of Nephrology, The Second Affiliated Hospital, Guangzhou Medical University, Guangzhou, China; 5 Department of Nephrology, Jiujiang No. 1 People’s Hospital, Jiujiang, China; 6 Department of Medical Statistics, Clinical Trials Unit, The First Affiliated Hospital, Sun Yat-Sen University, Guangzhou, China

**Keywords:** Peritoneal dialysis, High-density lipoprotein cholesterol, Diabetes, Peritonitis, Infection

## Abstract

**Background:**

Low levels of high-density lipoprotein cholesterol (HDL-C) and diabetes are common in patients undergoing peritoneal dialysis (PD). The aim of this study was to investigate the association between the coexistence of diabetes with a low level of HDL-C and the first episode of peritoneal dialysis-related peritonitis (PDRP) in patients with PD.

**Methods:**

We retrospectively investigated patients with PD from January 1, 2003, to May 31, 2020, in four PD centers. Patients with PD were divided into four groups: no comorbidities, low HDL-C only, diabetes only, and diabetes plus low HDL-C. The clinical and laboratory baseline data of the four groups were collected and compared. The association between diabetes coexisting with low HDL-C levels and the first episode of PDRP was analyzed by multivariate Cox regression analysis.

**Results:**

A total of 1013 patients with PD were included in our study. The mean age was 49.94 ± 14.32 years, and 597 (58.99%) patients were males. A total of 301 (29.7%) patients had their first episodes of PDRP, and low HDL-C levels coexisted with diabetes in 72 patients with PD. After adjusting for confounding factors, a low level of HDL-C coexisting with diabetes was significantly associated with the first episode of PDRP in our study (hazard ratio: 2.81, 95% CI 1.32 ~ 4.73, p = 0.005). The associations among HDL-C, diabetes and PDRP were consistent in the following subgroups: sex, age, and pre-existing CVD (all *P* interaction > 0.05).

**Conclusions:**

Patients with both diabetes and low HDL-C levels were at higher risk for PDRP in patients with PD.

**Supplementary Information:**

The online version contains supplementary material available at 10.1186/s13098-022-00832-x.

## Background

Peritoneal dialysis (PD) is an important renal replacement therapy. Peritoneal dialysis-related peritonitis (PDRP) is associated with mortality and technical failure in patients with PD [1, 2]. Diabetic nephropathy has risen in recent years in China [3]. Patients with diabetes are at increased risk for bacterial infection [4] Whether diabetes is associated with PDRP in patients with PD is controversial. In some previous studies, diabetes was identified as an independent risk factor for PDRP in patients with PD [5, 6]. In contrast, diabetes was not identified as an independent risk factor for PDRP in other previous reports [7] Dyslipidemia is common in patients with diabetes. It has been reported that a low level of high-density lipoprotein cholesterol (HDL-C) is a risk factor for infection in patients with diabetes [8].

Low levels of HDL-C are a manifestation of dyslipidemia in patients with PD and have been associated with mortality and cardiovascular disease (CVD) in patients with PD in many reports [9, 10]. HDL-C protects patients from serious infection, and low levels of HDL-C are also a risk factor for adverse outcomes in sepsis [11]. It has been reported that 50% of people with type 2 diabetes have low HDL-C concentrations [12]. Since diabetes was not necessarily an independent risk factor for PDRP, we presumed that diabetes coexisting with low levels of HDL-C might be associated with PDRP in patients with PD. In this study, we investigated the association diabetes coexisting low HDL-C levels with the first episode of PDRP in patients with PD.

## Methods

### Patients

Patients were recruited from four peritoneal dialysis centers in three provinces in China in this retrospective multiple-center study. Our study included adult patients aged ≥ 18 with PD recruited from January 1, 2003, to May 31, 2020. These patients received continuous ambulatory peritoneal dialysis (CAPD) with a standard glucose solution. Patients were excluded if they were on PD for < 3 months or had no lipid testing.

### Data collection and clinical definitions

The demographic and clinical characteristics, including age, sex, weight, height, blood pressure, history of smoking and alcohol use, pre-existing CVD, pre-existing stroke, residual urinary volume, use of statins, and laboratory test results, were recorded at baseline by at least two trained nurses. Laboratory characteristics included routine blood tests, biochemical tests, kidney and liver function tests, and lipid levels. These records were rechecked by at least two trained doctors.

Diabetes was defined as follows: (1) fasting plasma glucose ≥ 7.0 mmol/L, (2) 2 h plasma glucose ≥ 11.1 mmol/L during an oral glucose tolerance test (OGTT),o (3) glycated hemoglobin (HbA1c) ≥ 6.5%, (4) diabetes symptoms plus random plasma glucose ≥ 11.1 mmol/L, or (5) the use of glucose-lowering drugs. Diabetes symptoms are polydipsia, polyuria, polyphagia and unexplained weight loss. If patients had no symptoms of diabetes and only once had hyperglycemia, criteria 1 to 3 were confirmed by repeated testing [13]. A low level of HDL-C was defined as < 1.0 mmol/L according to Chinese guidelines on the prevention and treatment of dyslipidemia in adults [14].

A diagnosis of the first episode of PDRP was made if the patient had at least two of the following criteria according to the 2017 ISPD guidelines [15]: (1) abdominal pain with or without cloudy peritoneal dialysis effluent and with or without fever, (2) total leukocyte count of the dialysis effluent ≥ 100 × 10^6^ cells/L, with more than 50% polymorphonuclear cells in the differential count, and (3) positive Gram staining or culture of peritoneal dialysis effluent.

### Outcomes and follow-up

The outcome of our study was the first episode of PDRP. Patients with PD routinely returned to each center and were tested every 3 months in each center. If patients did not return, they received telephone interviews. September 1, 2020, was the final follow-up date in this study. Patients without PDRP were followed up until death or PD cessation. The time at which patients received hemodialysis, kidney transplantation, transferred care to another dialysis center, or were lost to follow-up were also recorded.

### Statistical analysis

Quantitative data are presented as the mean ± standard deviation (SD) or median (interquartile range [IQR]) after testing for normality. Nominal data are described as percentages. Baseline patient characteristics were compared for each group by chi-squared, one-way ANOVA, or Kruskal–Wallis tests. Univariate Cox regression analysis was used for the preliminary exploration of variables to estimate hazard ratios (HRs) with 95% confidence intervals (95% CIs) for the first episode of PDRP.

Survival curves and the time to peritonitis were calculated using the Kaplan–Meier method. Multivariate Cox regression analysis was conducted to examine the association between diabetes coexisting with low HDL-C levels and the first episode of PDRP using the following models: Model 1, unadjusted; Model 2, Model 1 plus demographic and clinical characteristics; and Model 3, Model 2 plus laboratory variables and medications. Since other outcomes, such as death, renal transplantation, transferred to hemodialysis and transfer to other centre, may influence standard regression test results for peritonitis, further analyses were done taking the competing risk of these outcomes into consideration. Competing-risks regression analysis was performed and sub-hazard ratios (SHR) was presented using Fine and Gray's competing risk regression analysis. Subgroups of sex, age, and history of pre-existing CVD were also analyzed. A p value across groups and the interactions between sex, age, and history of pre-existing CVD and PDRP were examined. The results are presented as HRs and 95% confidence intervals (95% CI). P values were two-sided, and P < 0.05 was considered to be statistically significant. All statistical analyses were performed with SPSS statistical software (version 21.0; Chicago, IL, USA), R (http://www.R-project.org), EmpowerStats software (www.empowerstats.com, X&Y Solutions, Inc., Boston, MA, USA), and Stata software (version 16; StataCorp, USA).

## Results

### Clinical baseline data of enrolled patients

**Fig. 1:**
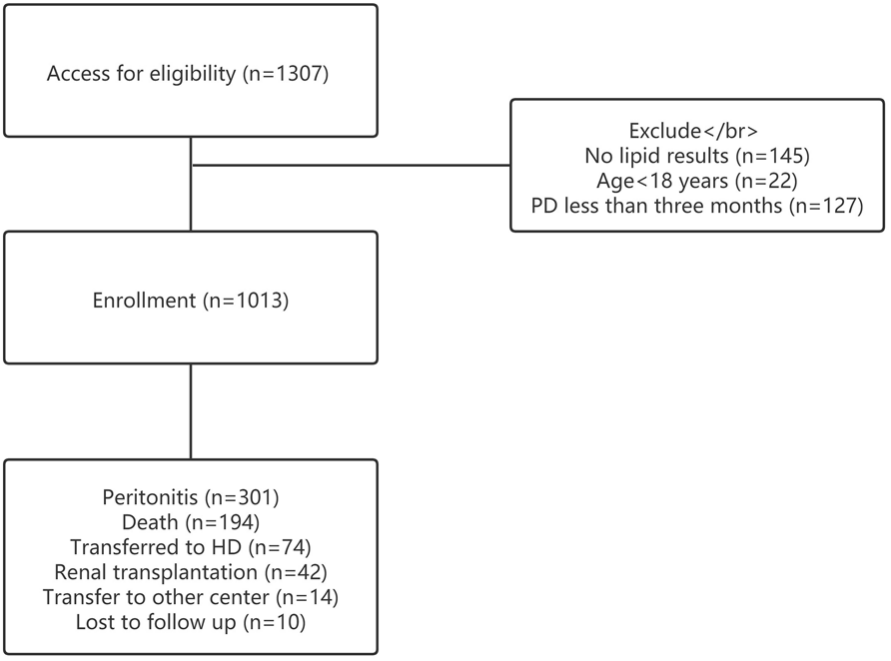
Process of patients inclusion

A total of 1307 patients were included in our study; they were recruited from four peritoneal dialysis centers from January 1, 2003, to May 31, 2020. The patients with diabetes in our study had type 2 diabetes. None of the patients were diagnosed with type 1 diabetes. A total of 145 patients were excluded due to no available lipid level results. Twenty-two patients were excluded because they were younger than 18 years. A total of 127 patients were excluded because the duration of follow-up was < 3 months. The remaining 1013 patients were analyzed in our study (Fig. 1).

Patients were divided into four groups according to their HDL-C levels and the presence of diabetes: Group 0 (no comorbidity), Group 1 (low HDL-C only), Group 2 (DM only), and Group 3 (both DM and low HDL-C). A total of 472 patients (46.6%) were assigned to Group 0, 374 patients (36.9%) were assigned to Group 1, 95 patients (9.4%) were assigned to Group 2, and 72 patients (7.1%) were assigned to Group 3. A total of 167 patients (Group 2 plus Group 3, 16.5%) had diabetes. A total of 446 patients (Group 1 plus Group 3, 44.0%) had low HDL levels. The baseline demographics, clinical and laboratory characteristics, and medications are summarized in Table 1. The mean age was 49.94 ± 14.32 years old, and 597 patients (58.99%) were male. A total of 193 patients (19.05%) had a history of current smoking, 71 (7.01%) had a history of current alcohol consumption, and 39 (3.85%) had pre-existing stroke. A total of 104 patients (10.27%) had pre-existing CVD, and 145 (14.31%) received statin therapy prior to PD. There were no differences in serum calcium or 24 h urine volume among the four groups. Age and BMI were higher in the DM plus low HDL-C level group. The incidence of pre-existing stroke and pre-existing CVD was also higher in the DM plus low HDL-C level group. WBC and triglyceride levels were higher in the DM plus low HDL-C level group. Serum potassium was lower in the DM plus low HDL-C level group.

**Table 1:** Baseline demographic characteristics, medications, and laboratory parameters

Variables^a^	Total (n = 1013)	No comorbidity (n = 472)	Low HDL-C (n = 374)	DM (n = 95)	DM plus low HDL-C (n = 72)	P-value
Age (years)^b^	49.94 ± 14.32 (n = 1013)	47.89 ± 14.04	47.98 ± 14.20	59.67 ± 9.93	60.74 ± 11.56	** < 0.001**
Male [n (%)]	597 (59.0%, n = 1013)	241(51.1%)	267 (71.4%)	51 (53.7%)	38 (53.5%)	** < 0.001**
Body mass index (kg/m^2^)^b^	22.81 ± 3.23 (n = 994)	22.04 ± 2.98	23.34 ± 3.36	23.76 ± 3.14	23.91 ± 3.10	** < 0.001**
Residual urine volume (mL)^c^	800.00 (400.00–1245.00, n = 619)	750.00 (400.00–1200.00)	810.00 (450.00–1338.75)	700.00 (487.50–1200.00)	800.00 (325.00–1045.00)	0.245
Current smoking [n (%)]	193 (19.1%, n = 1013)	72 (15.3%)	89 (23.8%)	19 (20.0%)	13 (18.1%)	**0.019**
Current alcohol consumption [n (%)]	71 (7.0%, n = 1013)	21 (4.5%)	39 (10.4%)	7 (7.4%)	4 (5.6%)	**0.002**
Pre-existing stroke [n (%)]	39 (3.9%, n = 1013)	6 (1.3%)	15 (4.0%)	9 (9.5%)	9 (12.5%)	** < 0.001**
Pre-existing CVD [n (%)]	104 (10.3%, n = 1013)	21 (4.5%)	24 (6.4%)	30 (31.6%)	29 (40.3%)	** < 0.001**
WBC (10^9^/L)^b^	6.37 ± 2.30 (n = 1013)	5.95 ± 2.10	6.53 ± 2.53	6.80 ± 2.09	7.57 ± 2.04	** < 0.001**
Hemoglobin (g/L)^b^	97.07 ± 23.36 (n = 1009)	97.83 ± 24.26	93.05 ± 22.67	105.35 ± 22.32	101.85 ± 17.59	** < 0.001**
Serum albumin (g/L)^b^	34.49 ± 5.65 (n = 1008)	34.83 ± 5.38	35.06 ± 5.97	31.81 ± 4.72	32.80 ± 5.66	** < 0.001**
AST (U/L)^c^	17.00(13.00–22.00, n = 918)	17.00 (13.00–22.00)	16.00 (12.00–22.00)	19.00 (15.50–24.50)	18.50 (14.00–24.25)	** < 0.001**
ALT (U/L)^c^	13.00 (9.00–20.00, n = 918)	13.00 (9.00–20.00)	14.00 (8.00–20.00)	15.00 (11.00–21.00)	12.00 (8.00–16.25)	**0.024**
Cholesterol (mmol/L)^b^	4.42 ± 1.31 (n = 1009)	4.63 ± 1.25	3.92 ± 1.09	5.00 ± 1.41	4.90 ± 1.73	** < 0.001**
Triglyceride (mmol/L)^c^	1.30 (0.94–1.88, n = 1009)	1.13 (0.82–1.51)	1.53 (1.11–2.17)	1.20 (0.91–1.79)	1.92 (1.24–2.79)	** < 0.001**
Low-density lipoprotein cholesterol (mmol/L)^b^	2.67 ± 0.97 (n = 1009)	2.76 ± 0.98	2.47 ± 0.87	2.91 ± 1.17	2.74 ± 0.95	** < 0.001**
Serum calcium (mmol/L)^b^	2.12 ± 0.28 (n = 1013)	2.13 ± 0.28	2.09 ± 0.31	2.11 ± 0.23	2.16 ± 0.24	0.077
Serum phosphorus (mmol/L)^b^	1.66 ± 0.67 (n = 1013)	1.65 ± 0.59	1.77 ± 0.70	1.50 ± 0.48	1.43 ± 1.04	** < 0.001**
Serum potassium (mmol/L)^b^	4.11 ± 0.85 (n = 1013)	4.12 ± 0.83	4.16 ± 0.81	4.12 ± 0.96	3.82 ± 0.97	**0.019**
IPTH (pg/mL)^c^	31.40 (12.25–93.56, n = 552)	32.45 (12.05–106.45)	32.90 (14.50–98.00)	31.40 (14.20–97.35)	17.10 (5.98–63.10)	0.073
Statins [n (%)]	145 (14.3%, n = 1013)	56 (11.9%)	39 (10.4%)	33 (34.7%)	17 (23.6%)	** < 0.001**
Peritonitis [n (%)]	301 (29.7%, n = 1013)	153 (32.4%)	87 (23.3%)	31 (32.6%)	30 (41.7%)	**0.002**

Statistically significant results are indicated in bold

*HDL-C* high-density lipoprotein cholesterol, *DM* diabetes mellitus, *WBC* white blood cell, *CVD* cardiovascular disease, *ALT* alanine aminotransferase, *AST* aspartate aminotransferase, *iPTH* intact parathyroid hormone

^a^Data are expressed as number (%) unless otherwise indicated

^b^Data are expressed as mean ± standard deviation

^c^Data are expressed as median (interquar the range)

### ***Risk factors for the first episode of PDRP in ***patients*** with PD***

As shown in Table 2, after univariate Cox regression, diabetes, pre-existing stroke, pre-existing CVD, statins, hemoglobin, serum albumin, HDL-C, low HDL-C group, and diabetes plus low HDL-C group were associated with the first episode of PDRP in patients with PD.

**Table 2:** Risk factor associated with the first episode of peritonitis

Variables	HR (95% CI)	P-value
Age (decades)	1.0 (0.9, 1.1)	0.991
Gender		
Male	1.0	
Female	1.1 (0.9, 1.4)	0.417
BMI (kg/m^2^)	1.0 (1.0, 1.0)	0.953
Residual urine volume (mL)	1.0 (1.0, 1.0)	0.189
Current smoking	1.3 (1.0, 1.8)	0.066
Current alcohol consumption	1.2 (0.8, 2.0)	0.338
DM	1.8 (1.4, 2.4)	** < 0.001**
Pre-existing stroke	2.0 (1.2, 3.3)	**0.009**
Pre-existing CVD	1.8 (1.3, 2.5)	** < 0.001**
Statins	1.9 (1.4, 2.6)	** < 0.001**
WBC (10^9^/L)	1.1 (1.0, 1.1)	0.130
Hemoglobin (g/dL)	1.0 (0.9, 1.0)	**0.023**
Serum albumin (g/dL)	0.7 (0.6, 0.9)	**0.007**
AST (U/L)	1.0 (1.0, 1.0)	0.495
ALT (U/L)	1.0 (1.0, 1.0)	0.578
Cholesterol (mmol/L)	0.9 (0.9, 1.0)	0.253
Triglyceride (mmol/L)	0.9 (0.8, 1.1)	0.301
High-density lipoprotein cholesterol (mmol/L)	1.3 (1.1, 1.6)	**0.002**
Low-density lipoprotein cholesterol (mmol/L)	0.9 (0.8, 1.0)	0.180
Serum calcium (mmol/L)	0.9 (0.6, 1.4)	0.747
Serum phosphorus (mmol/L)	0.8 (0.7, 1.0)	0.081
IPTH (pg/mL)	1.2 (1.0, 1.4)	**0.017**
Serum potassium (mmol/L)	1.1 (1.0, 1.3)	0.088
GROUP		
No comorbidity	1.0	
Low HDL-C	0.8 (0.6, 1.0)	**0.037**
DM	1.4 (1.0, 2.1)	0.072
DM plus LOW HDL-C	1.9 (1.3, 2.8)	**0.002**

Statistically significant results are indicated in bold

*HR* hazard ratio, *CI* confidence interval, *WBC* white blood cell, *CVD* cardiovascular disease, *ALT* alanine aminotransferase, *AST* aspartate aminotransferase, *iPTH* intact parathyroid hormone, *HDL-C* high-density lipoprotein cholesterol, *DM* diabetes mellitus

### Observational period and outcome

The overall follow-up period was 403,213 patient-months, with a median period of 32.0 (4.0–211.0) months per patient. At the end of the study, 194 (19.15%) patients had died, 74 (7.31%) patients were transferred to hemodialysis, 42 (4.15%) patients received renal transplantation, 14 (1.38%) patients were transferred to other centers, and 10 (0.99%) patients were lost to follow-up. A total of 301 (29.7%) patients had their first episode of PDRP, and the incidences of a first episode of PDRP in Groups 0, 1, 2 and 3 were 32.4%, 23.3%, 32.6% and 41.7%, respectively.

### ***Associations between low HDL-C levels and diabetes and the first episode of PDRP in ***patients*** with PD***

**Fig. 2:**
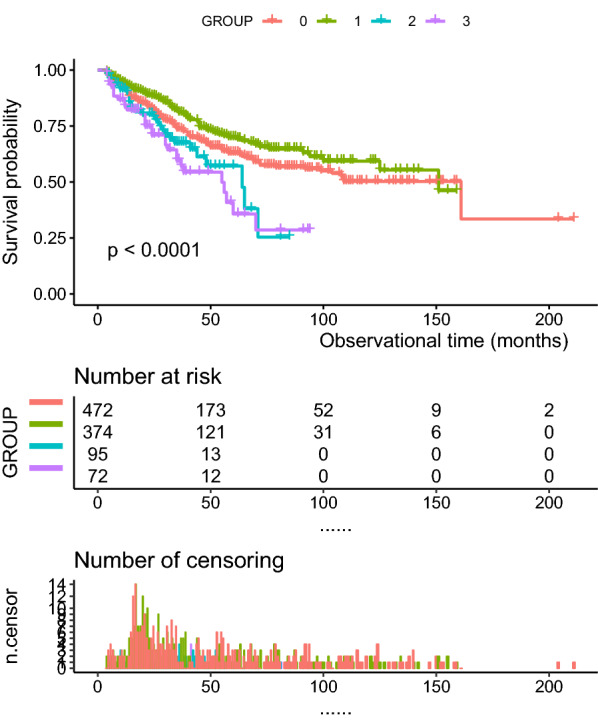
Kaplan–Meier curve of overall peritonitis-free survival

**Fig. 3:**
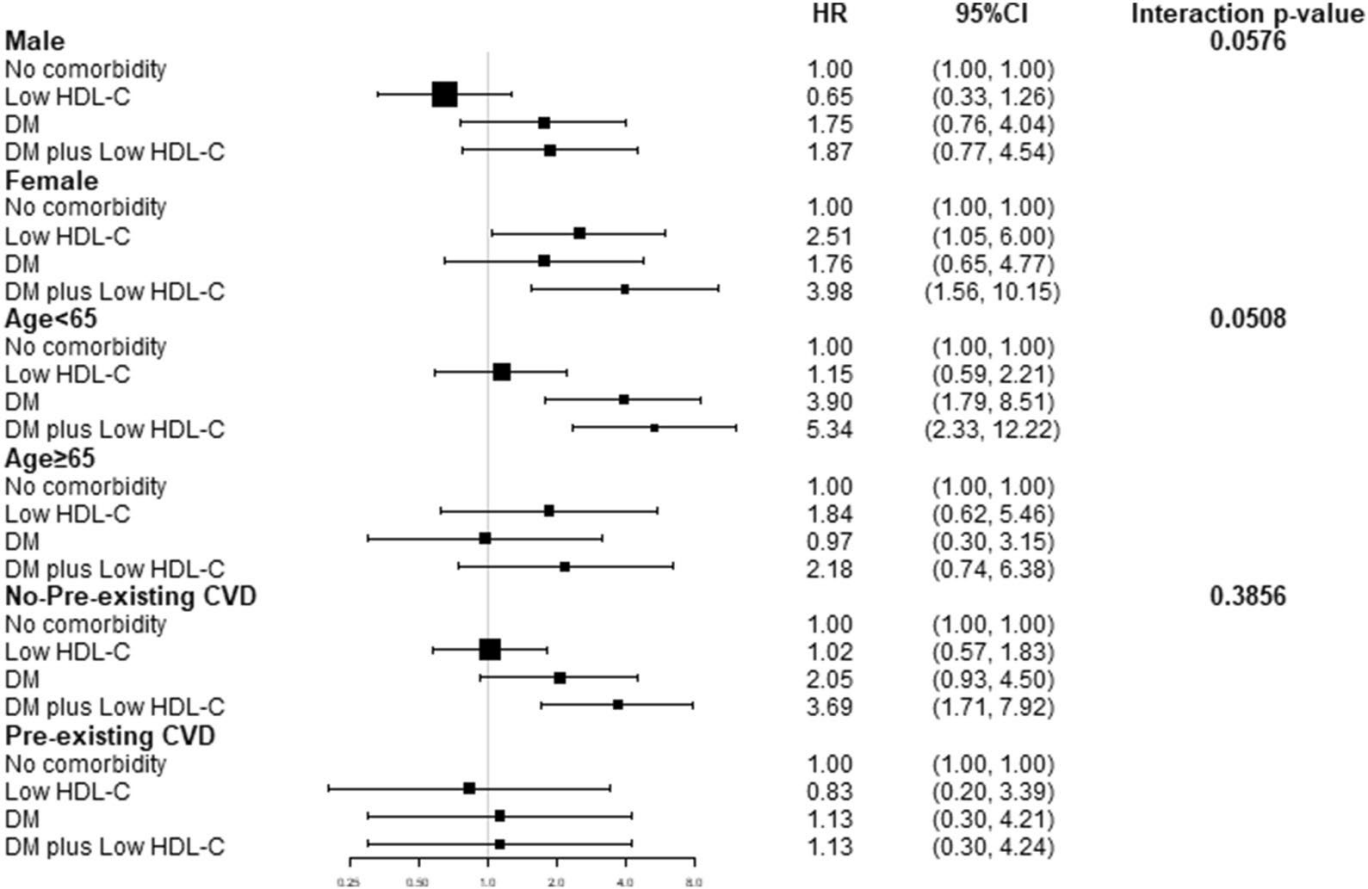
Subgroup analysis of gender, age and pre-existing cardiovascular disease

In the survival analyses, the overall peritonitis-free survival of patients in the DM plus low HDL group declined significantly faster than that in the other groups (p < 0.0001, Fig. 2). We validated the Cox regression using Stata 16, and the global test p value was 0.5005. The associations between low HDL-C levels and diabetes and PDRP are presented in Table 3. After adjusting for sex, age, BMI, current smoking, pre-existing CVD, pre-existing stroke, statins, and laboratory tests (Table 3), compared to Group 0, Groups 1, 2, and 3 had a 0.787 (95% CI 0.63 ~ 1.83), 1.83 (95% CI 0.98 ~ 3.41), and 2.81 (95% CI 1.32 ~ 4.73) higher risk for PDRP, respectively (using Model 3). Diabetes plus a «low level of HDL was significantly associated with a higher risk (HR = 2.81, 95% CI 1.32 ~ 4.73, p = 0.005) for the first episode of PDRP. In the competing risk model analysis, DM plus Low HDL-C (Group3) were significant for the first episode of peritonitis (sHR 1.99, 95% CI 1.07 ~ 3.73, p = 0.031), death (p = 0.001) and renal transplantation (p = 0.001), but they were not significantly different for transferred to hemodialysis (p = 0.187), or transfer to other centre (p = 0.873). An addional figure file shows the CIF curve [see Additional file 1]. The subgroups of sex, age, and pre-existing CVD are shown in Fig. 3. The p values for the interactions were > 0.05 for subgroups by sex (p = 0.0576), age (p = 0.0508) and pre-existing CVD (p = 0.3856).

**Table 3:** Association among DM and Low HDL-C and the first episode of peritonitis

	Model 1^**a**^		Model 2^**b**^	P-value	Model 3^**c**^	P-value
	HR (95% CI)	P-value	HR (95% CI)		HR (95% CI)	
No comorbidity	1.0 (ref.)		1.0		1.0	
Low HDL-C	**0.76 (0.58, 0.98)**	**0.037**	**0.74 (0.56, 0.97)**	**0.032**	1.07 (0.63, 1.83)	0.787
DM	1.43 (0.97, 2.11)	0.072	1.32 (0.86, 2.01)	0.202	1.83 (0.98, 3.41)	0.057
DM plus low HDL-C	**1.86 (1.25, 2.76)**	**0.002**	**1.69 (1.09, 2.62)**	**0.020**	**2.81 (1.32, 4.73)**	**0.005**

Statistically significant results are indicated in bold

*HR* hazard ratio, *CI* confidence interval, *HDL-C* high-density lipoprotein cholesterol, *DM* diabetes mellitus

^a^Unadjusted

^b^Model 1 plus age, sex, body mass index, current smoking, pre-existing stroke, pre-existing cardiovascular disease

^c^Model 2 plus hemoglobin, serum albumin, cholesterol, triglyceride, low-density lipoprotein, intact parathyroid hormone, statins and serum potassium

## Discussion

The rate of diabetes in patients with PD was 16.5% in our study. This result is similar to previous literature reports. The cause of ESRD was diabetic nephropathy in 16.4% of patients in China [16]. We found that 44.0% patients had low HDL serum levels in our study. A total of 7.1% of patients with diabetes also had low HDL-C levels in our study. We found that comparing to no comorbidities, diabetes and concurrent low HDL-C levels were more associated with the first episode of PDRP in patients with PD than either diabetes or low HDL levels alone in our study.

It has been reported that the PDRP rate is higher in DM patients than in non-DM patients [17]. Diabetes was indicated as a risk factor for PDRP in previous reports [18, 19]. Diabetes alters the immunity of peritoneal defenses, such as leukocyte adherence, chemotaxis, and phagocytosis. Diabetes also interferes with the migration of phagocytic cells into the peritoneum and suppresses the phagocytic activity of resident peritoneal macrophages [5]. Not all studies support this conclusion. Some studies found that diabetes was not an independent risk factor for PDRP [20, 21]. Hyperglycemia was reported to be a predictor of the risk for tunneled catheters and existing infections but not for peritoneal infections [22]. Low HDL-C levels were observed in diabetes patients. A lack of apo AI and apo AII and increased clearance of HDL are the main reasons for low HDL-C levels in diabetes [23]. HDL plays an important role in fighting infection in many ways. HDL binds and neutralizes gram-negative bacterial lipopolysaccharide (LPS) and gram-positive bacterial lipoteichoic acid (LTA). HDL inhibits the expression of adhesion molecules that is induced by proinflammatory cytokines, such as V-CAM-1, ICAM-1, and E-selectin, after inflammation. HDL may also prevent monocyte activation and recruitment. As a result, the inflammatory response decreases after sepsis [11]. HDL limits oxidation by decreasing ROS production and inhibiting LDL oxidation. Low HDL levels lead to a decrease in antioxidation, which exacerbates damage from infection [24]. Low HDL levels are a risk factor for foot infection in diabetic foot osteomyelitis [25]. Low HDL levels were also associated with parasitic disease and Mycobacterium tuberculosis infection in patients with diabetes [26, 27]. Low HDL levels were associated with periodontal infection in patients with diabetes [28]. All these reports demonstrate that diabetes coexisting with low HDL-C levels is associated with infection. HDL-C binds to pathogenic microorganisms and reduces inflammatory damage in diabetes. PDRP is a typical bacterial infection in patients with PD. Diabetes plus low HDL-C levels increased the risk for PDRP in patients with PD in our study. The K-M curves confirmed this result. It is therefore important to maintain normal serum HDL levels in patients with diabetes with PD.

PD patients usually show increased levels of triglycerides (TGs), cholesterol (CHOL), and low-density lipoprotein cholesterol (LDL-C) and decreased levels of HDL-C [29]. Since disorders of HDL-C are associated with severe infection and exaggerate the systemic inflammatory response [30–32], we analyzed the association between HDL-C levels and PDRP in patients with PD. We found that low HDL-C levels were not independently associated with PDRP in PD patients in our study. The reason might be that dyslipidemia is a complicated process in patients with PD. Disorders of TG, CHOL and LDL-C also participate in the pathological process of PDRP. Low HDL-C levels alone were not enough to be an independent risk factor for PDRP in our study.

In previous studies, dyslipidemia and poor glycemic control were reported to be risk factors for ESRD and mortality in young patients and women [33–35]. HDL-C was inversely associated with the left ventricular mass index in patients with PD [36]. Subgroups of age, sex, and history of cardiovascular disease were analyzed in our study. We found that the association between DM plus low HDL-C levels and PDRP was not affected by age, sex, or history of cardiovascular disease after adjusting for age, sex, body mass index, current smoking, pre-existing stroke, pre-existing CVD, statins and laboratory tests except for the subgroup variable. We confirmed that DM plus low HDL levels is an independent risk factor for PDRP in patients with PD.

Our study has several limitations. First, although the study was a multicenter study with more than 1000 patients, the patients enrolled in our study had type 2 diabetes. We did not analyze patients with type 1 diabetes. Second, our study only looked at the association between DM plus a low level of HDL-C and PDRP. We could not determine the causality relationship between DM plus low HDL-C and PDRP. Third, the TG, CHOL, and LDL-C levels were associated with HDL-C levels. Thus, we should detect the detailed relationships among TGs, CHOL, LDL-C, and HDL-C in patients with PD and evaluate the effects of the interactions between HDL-C and other lipids on PDRP.

## Conclusions

This study showed that comparing to no comorbidities, diabetes and concurrent low HDL-C levels were more associated with the first episode of PDRP in patients with PD than either diabetes or low HDL levels alone in our study. It is important to maintain normal levels of HDL-C in patients with diabetes with PD to avoid PDRP.

## Supplementary Information

**Additional file 1:** Cumlative incidence function curve of peritonitis.

## Data Availability

The datasets used and analysed during the current study are available from the corresponding author on reasonable request.

## Abbreviations

ALT: Alanine amiotransferase
ANOVA: Analysis of variance
AST: Aspartate transaminase
CHOL: Cholesterol
CI: Confidence intervals
CVD: Cardiovascular disease
DM: Diabetes mellitus
ESRD: End-stage renal disease
HbA1c: Glycosylated hemoglobin, type A1c
HDL: High-density lipoprotein
HDL-C: High-density lipoprotein-cholesterol
HR: Hazard ratio
IQR: Interquartile range
K-M: Kaplan–Meier
LDL: Low-density lipoprotein
LDL-C: Low-density lipoprotein-cholesterol
OGTT: Oral glucose tolerance test
PD: Peritoneal dialysis
PDRP: Peritoneal dialysis-related peritonitis
SD: Standard deviation
TG: Triglyceride
WBC: White blood cell
