# Supplementary figures and images for: The association between diabetes coexisting with low levels of high-density lipoprotein cholesterol and peritoneal dialysis-related peritonitis
Source: Diabetol Metab Syndr. 2022 Apr 29;14:60. doi: 10.1186/s13098-022-00832-x (PMC9052536; doi:10.1186/s13098-022-00832-x)

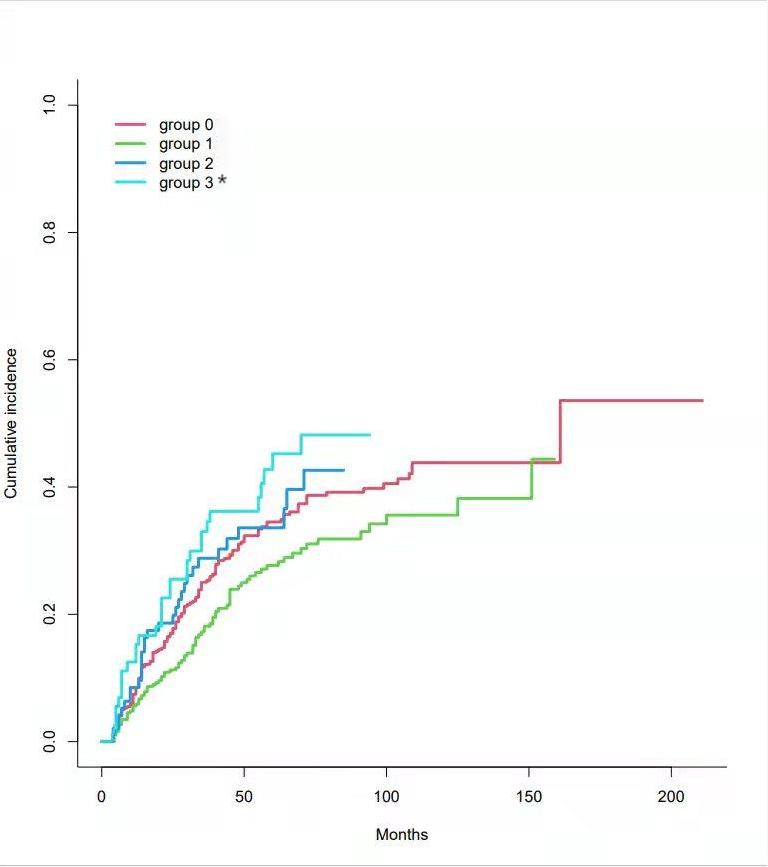

Supplement: Supplementary file 1 — Additional file 1: Cumlative incidence function curve of peritonitis. [file 13098_2022_832_MOESM1_ESM.jpg]
